# Surface electromyography during physical exercise in water: a systematic review

**DOI:** 10.1186/2052-1847-6-15

**Published:** 2014-04-15

**Authors:** Antonio Ignacio Cuesta-Vargas, Carlos Leonardo Cano-Herrera

**Affiliations:** 1 Departamento de Psiquiatria y Fisioterapia, Instituto de Biomedicina de Malaga (IBIMA), Grupo de Clinimetria (AE-14), Universidad de Málaga, Andalucía Tech, Facultad de Ciencias de la Salud, Av/ Arquitecto Peñalosa s/n (Teatinos Campus Expansion), 29009 Malaga, Spain; 2 School of Clinical Sciences of the Faculty of Health at the Queensland University of Technology, Brisbane, Australia; 3 Universidad de Málaga, Andalucía Tech, Facultad de Ciencias de la Salud, Av/ Arquitecto Peñalosa s/n (Teatinos Campus Expansion), 29009 Málaga, Spain

**Keywords:** Electromyography, Aquatics, Hydrotherapy, Review

## Abstract

**Background:**

Aquatic exercise has been widely used for rehabilitation and functional recovery due to its physical and physiological benefits. However, there is a high variability in reporting on the muscle activity from surface electromyographic (sEMG) signals. The aim of this study is to present an updated review of the literature on the state of the art of muscle activity recorded using sEMG during activities and exercise performed by humans in water.

**Methods:**

A literature search was performed to identify studies of aquatic exercise movement.

**Results:**

Twenty-one studies were selected for critical appraisal. Sample size, functional tasks analyzed, and muscles recorded were studied for each paper. The clinical contribution of the paper was evaluated.

**Conclusions:**

Muscle activity tends to be lower in water-based compared to land-based activity; however more research is needed to understand why. Approaches from basic and applied sciences could support the understanding of relevant aspects for clinical practice.

## Background

Exercise in the aquatic environment has been widely used for rehabilitation and functional recovery due to its physical and physiological benefits [1]. People who cannot tolerate the mechanical stress of exercise in a dry environment can benefit from aquatic exercise and achieve physical and physiological responses that will provide benefits to their health or physical condition.

Physiotherapists have recommended the use of exercise in water due to the advantages offered by hydrostatic pressure, drag forces, and propulsion [2]. The buoyant force acting in the opposite direction to the force of gravity and drag forces in the opposite direction to the movement of the body in water cause muscle activation to be different in intensity and degree of participation depending of the activities and exercises used. For this reason it would be interesting to know the degree of muscle activation in water during various activities and exercises in order to select the appropriate rehabilitation program in water. Likewise, there is little understanding of muscle activity in water activities for use in physical activities in water and sports (aqua-fitness, recreational swim…), which are very useful for maintaining or improving the physical condition without placing excessive load on the spine and extremities [3].

The effects of aquatic therapy is often used in pediatrics [4], orthopedics [5], rheumatology [6], neurology [7] and many others [8]. Aquatic therapy includes a large hands-on component, especially in neurological rehabilitation. In these populations treatment is varied and complex and aquatic therapy is usually only a minor component. Nonetheless, this might have an important place in the long-term effect of rehabilitation where any treatment is small in measurable terms. Quantifying the effect of aquatic therapy has, as a consequence, not gained sufficient attention. For this reason, the first step toward development of an effective therapy program of water-based exercise will be to gain a better understanding of muscle activity during exercise in water. In the literature on aquatic exercise and activity there is a high variability in reporting on the muscle activity from surface electromyography [sEMG] signals [9]. This variability is due to various factors such as differences in the pool depth and water temperature, water activity familiarization, regulation of exercise intensity, and so on, and some conclusions about the level of muscle activation and recruitment patterns are contradictory.

Measuring muscle activity during exercise in the water is difficult and often not attempted, as most instruments are not designed for this type of environment and are therefore are often unreliable or not valid. For example, quantitation of muscle activity by techniques of electromyography [EMG] during locomotion in water is challenging due to the difficulty of preventing the inferred water in the recording of the electrical signal of a muscle and, for reasons of safety, with respect to the immersion of the electrical components in water [e.g. electrocution]. In addition there could be some minor issues related to the EMG signal, the most probable reason for this is that the weightlessness or buoyancy effect on the neuromuscular system is not yet fully explained [9].

This review aims to assess the effectiveness of surface EMG to measure muscle activity during aquatic exercise and compare its use to similar land based exercise situations.

## Methods

### Data sources

A literature search was performed to identify relevant studies about aquatic therapy. PEDro, CINALH [ovid], PUBMED, EMBASE, AMED, AgeLine, the Cochrane Library, and SPORTDiscus databases were examined. The databases were searched using combinations of the keywords and search limits (1997–2013), which are presented in Table 1. The manuscript adheres to the PRISMA guidelines for reporting systematic reviews.

**Table 1:** Keywords and limits of systematic review

	
**Keywords:**	
Hydrotherapy	Neuromuscular
Aquatic	Electromyography
Water	EMG
Dry	
**Limits:**	
Humans	
English and Spanish languages	
Published in the previous 15 years (1997–2013)	

### Study selection or eligibility criteria

The studies that were selected were those that made a comparison of neuromuscular activity in human subjects who performed an aquatic exercise and the same or similar land-based exercise.

### Study appraisal and synthesis methods

The final selection was made based on the abstract or title. We excluded and removed case-reports, studies that did not make comparisons with activity or land-based exercise and those that made comparisons of how to use local or immersion electrodes in water. Two independent reviewers completed the quality appraisal, with disagreements resolved by consensus. The studies were critically appraised using the Spanish Critical Appraisal Skills Programme [CASPe] tool for comparison studies; more details could be checked in the site http://www.redcaspe.org/moodle/. Appraisal criteria were not applied to the conference proceedings or abstract-only reports because their brevity limited the provision of methodological detail. Two independent reviewers [CV & CH] carried out the critical appraisal.

## Results

**Figure 1:**
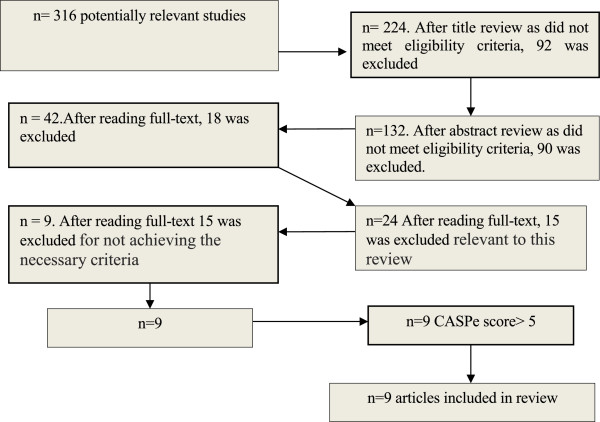
Flow-chart displaying selection of studies.

Three hundred sixteen articles were found in electronic search and one hundred thirty two were examined after selection based on the title and abstract. Forty-two relevant articles were found in the main databases. Twenty-four original subsequent studies were examined after selection based on reading full text and 15 were excluded for not achieving the necessary criteria [Figure 1]. There were no irresolvable disagreements between authors. All 9 studies scored greater than five. This CASPe tool has not been an elimination criterion. The studies included in this review share common threats to validity as most studies score negatively in the same areas.

The results of this review are given in Table 2 in chronological order. The Table 2 shown a summary of the differences between the aquatic and land exercises/activities, each study present differences task, and muscle, however the statistical analysis to assess the performance of EMG peak values were heterogeneous, but due to the heterogeneity of EMG parameters, this information was included with more details under clinical contribution in the Table 2.

**Table 2:** Reviewed papers about electromyography of physical exercise in water

**Study**	**Subjects**	**Tasks [in water]**	**Comparison [land-based]**	**Muscles**	**Clinical contribution**
[Castillo-Lozano et al., 2013] [10]	16 healthy adults	3 arm elevation movements [flexion, abduction, and scaption] through 0° to 90°	The same exercises	ES, UT, PM, AD, MD, LD	Muscle activity levels were significantly lower in water compared with dry land at 30°/sec and 45°/sec but significantly higher at 90°/sec
[Cuesta-Vargas et al., 2013] [11]	10 healthy subjects	Lower limb and trunk muscles MVC and the STS task	The same MVC and STS task	VM, RF, BF, TA, GM, SOL, RA, ES	Muscle activity was significantly lower on water than land-based signals by MVC from VM, RF, BF, TA, GM and SOL. The muscle activity was higher in water for RA and ES
[Cuesta-Vargas et al., 2013] [12]	Ten healthy subjects	MVC and TUG	The same exercises	RF, BF, TA, GM, SOL, RA, ES	The muscle activation of the trunk and the lower limb [VM RF, BF, TA, GM and SOL] were lower in water compared to dry land, when performing a TUG test
[Bressel et al., 2011] [13]	11 physically active young males	Abdominal hollowing, abdominal bracing and pelvic tilts	The same exercises	RA, EO, LA, MT, ES	EMG signals for all muscles were lower for all exercises performed in water than on land, except ES, which had the same during mediolateral pelvic tilts
[Silvers and Dolny, 2011] [14]	12 recreational young male runners	MVC tests of each muscle tested	The same MVC tests	VM, RF, BF, TA, GM	There were no differences in EMG signals between environments
[Alberton et al., 2011] [15]	12 physically active young and healthy women	Stationary running at submaximal intensities and at maximal velocity	The same exercises	RF, VL, ST, BF	At submaximal intensities EMG signals were lower in water, but at maximal effort were similar between environments
[Pinto et al., 2010] [16]	9 healthy young women	MVC tests of elbow flexion and extension, and for hip flexion and extension	The same MVC tests	BB, TB, RF, BF	There were no differences in EMG signals between environments
[Masumoto et al., 2009] [17]	7 healthy young subjects	Deep water running [DWR] at three levels of intensity	Treadmill running at three levels of intensity	RF, BF, TA, GA	TA and GA EMG signals during DWR were lower than during treadmill running at all RPE conditions. But RF, BF EMG signals were similar in both environments at all RPE conditions
[Barela and Duarte, 2008] [18]	10 elderly individuals	Walking at self-selected comfortable speeds	The same activity	TA, GM, VL, BF, TFL, RA, ES	The EMG activation patterns were different for all muscles [except GM, which was similar] between water and land
[Kaneda, Wakabayashi, Sato, Uekusa, & Nomura, 2008] [19]	9 healthy young males	DWR and walking at self-determined slow, moderate and fast paces	Walking at self-determined slow, moderate and fast paces	TA, SOL, GM, RF, VL, BF	The EMG signal of the BF during DWR was higher than during land water or water walking. Of the RF, during DWR was higher than during land walking, but similar to water walking. SOL, GM and VL EMG signals were lower during DWR
[Kaneda et al., 2009] [20]	9 healthy young males	DWR and walking at self-determined slow, moderate and fast paces	Walking at self-determined slow, moderate and fast paces	AL, GMa, GMe, RA, EO, ES	EMG signals were higher during DWR than during land walking and water walking
[Masumoto et al., 2008] [9]	9 healthy female older subjects	Walking on an underwater treadmill at three speeds and against a current	Walking on a treadmill at three different speeds	VM, RF, BF, TA, GL	EMG signals during walking in water were lower than when walking land-based at all speed conditions
[Chevutschi et al., 2007] [21]	7 young women	Walking at a comfortable speed	The same activity	SOL, RF, ES	SOL EMG signal was less during water walking than during land walking, RF EMG signal was similar in both environments, and the ES EMG signal was higher during water walking than during land walking
[Kaneda et al., 2007] [22]	9 healthy young males	DWR and walking at self-determined slow, moderate and fast paces	Walking at self-determined slow, moderate and fast paces	TA, SOL, GM, RF, BF	During DWR, SOL, GM and BF showed lower EMG signal than during land walking and water walking. During water walking, SOL and GAS showed lower activity than during land walking
[Shono et al., 2007] [23]	8 healthy older women	Walking on an underwater treadmill at a three different water-flow speeds	Walking on a treadmill a three different speeds	TA, GM, VM, RF, BF	At the same velocity, the EMG signals of TA, VM and BF were higher during water walking than during land walking, whereas those of RF and GM were similar in both environments
[Barela et al., 2006] [24]	10 healthy adults	Walking at self-selected comfortable speeds	The same activity	TA, GM, VL, BF, TFL, RA, ES	The EMG activation patterns were different for all muscles [except GM, which was similar] between water and land
[Masumoto et al., 2005] [25]	6 healthy young males	Walking backward on an underwater treadmill at three speeds and against a current	Walking backward on a treadmill at three different speeds	GMe, VM, BF, TA, GL, RA, ES	At all speeds, the EMG signals while walking in water [both with and without a current] were lower than when walking land-based [with the exception of the ES, which was higher during water walking]
[Masumoto et al., 2004] [26]	6 healthy young males	Walking on an underwater treadmill at three speeds and against a current	Walking on a treadmill at three different speeds	GMe, VM, BF, TA, GL, RA, ES	EMG signals during walking in water [both with and without a water current] were lower than walking land-based
[Miyoshi et al., Akai, 2004] [27]	15 healthy young males	Walking at comfortable, slower and faster speed	The same activities	GM, RF, TA, BF	With the increase of walking speed during water walking, the GM and BF activities were increased as compared to each EMG activity during land walking, but there were no changes in TA and RF EMG activities
[Pöyhönen and Avela, 2002] [28]	6 healthy young males	MVC test of plantar flexion	The same MVC test	SOL, GM	There were no differences in EMG signals between environments
[Pöyhönen et al., 2001] [29]	18 healthy young subjects	Maximal knee extension-flexion efforts against resistance in a sitting position	Maximum isometric and isokinetic force production sitting on an isokinetic dynamometer	ST, VM, VL, BF	Maximal activity during the knee extension and the activity at 90° of the VM and VL were lower in water than land-based. Maximal activity during the knee flexion of the ST and BF was higher in water than land-based, whereas, the activity at 90° was lower in water than land-based
[Kelly et al., 2000] [30]	6 healthy young males	Elevation of the arm in the scapular plane with neutral humeral rotation at three different speeds	The same activities	AD, MD, PD	At slow and medium speed, EMG signals were lower in water than land-based, however there were no differences between environments at fast speed
[Pöyhönen et al., 1999] [31]	12 healthy adults women	Maximal and submaximal isometric force production of the quadriceps in a sitting position	The same activities	VM, VL, BF	EMG signals were lower during maximal and submaximal contractions in water than land-based
[Fujisawa et al., 1998] [32]	8 healthy young males	Isometric exercises of shoulder flexion, abduction and rotation	The same exercises	AD, MD, PD, PM, LD	During flexion, abduction and maximal external rotation, the EMG signals were lower in water than land-based

Rectus abdominis [RA], external oblique abdominis [EO], lower abdominals [LA], multífidus [MT], erector spinae [ES], quadriceps – vastus medialis [VM], quadriceps – rectus femoris [RF], quadriceps – vastus lateralis [VL], bíceps femoris [BF], tibialis anterior [TA], gastrocnemius [GA], gastrocnemius medialis [GM], gastrocnemius lateralis [GL], semitendinosus [ST], bíceps brachii [BB], tríceps brachii [TB], tensor fasciae latae [TFL], soleus [SOL], adductor longus [AL], gluteus maximus [GMa], Gluteus medius [GMe], anterior deltoids [AD], middle deltoids [MD], posterior deltoids [PD], pectoralis mayor [PM], latissimus dorsi [LD], upper trapezius [UT].

## Discussion

Of the 24 articles selected, nine focused on comparing the same activity and/or land-based exercise and in water [10-13,18,21,24,27,30-32]. Although most of the studies describe limits on finding activities that were comparable in terms of kinetics and kinematics, in most muscle group’s activation was lower in water, especially in distal muscles. In cases where the pattern of activation was analyzed [18,24], it was determined that it was not possible to compare the activities as dry activation follows a different pattern to activation in water, probably due to the different depths at which each muscle group acts during running in water.

Eight studies focused on comparing different levels of intensity of the activity in the water [17,19,20,23,25,26,33]. The most frequently studied activities were walking and running.

Six of these studies analyzed *gait*[9,21,23,25]. The main problem with comparing muscle activation in water and on land-based exercise is that kinetic control [outgoing force] and kinematics [displacements and velocities] are different in each environment. However, in studies comparing walking in water and land-based there were some common findings. Activity of the rectus abdominis [RA], gluteus medius [GMe], quadriceps – vastus medialis [VM], biceps femoris [BF], tibialis anterior [TA], gastrocnemius lateralis [GL] muscles were shown by sEMG to be lower in water than land-based. Although it is not clear, it is speculated that water depth and exercise type influence muscle activation as there is less activity in distal muscles compared to proximal muscles.

Only one study examined the adaptation of muscle activity during incremental exercise [27], and as in other studies a lower activation of the distal muscles was found.

Walking backward was examined in a study and as for walking forward; values were lower in water than land-based [27]. Four studies analyzed *deep water running* [DWR] [17,18,20]. Only one study compared DWR on tape, finding lower activity of the distal muscles and similar activation of proximal muscles. These findings are consistent with study findings on walking with controlled levels of intensity, effort, and direction of motion.

The remaining DWR studies comparing walking in water with walking land-based [18,20] found discrepancies between the muscle activations, because these activities are not similar.

*Maximal Voluntary Contraction* [MVC] is the most common form of normalizing EMG data for comparison between individuals. Although it is a standardized method for dry exercises, it is unclear whether the EMG data recorded should be normalized for water from the dry-exercise data [34]. In this review three studies analyzed MVC land-based and in water and found that the environment did not affect the value, provided that the control of the muscle action was similar [14,16]. With regard to anatomical regions, two studies examined the knee [29,31], two the shoulder [30,32], and one, the lumbar region [13]. But the most remarkable aspect of these studies is that although they considered less functional activities, control of execution of the land-based exercise and in water with speed control [30] or by means of force projection [23], allowed similar activation to be found both land-based and in water with the same exercises.

In the last years there has been a great deal of research of surface EMG in the water. It seems that EMG during MVC is lower when performed in water compared to the dry land. It is unclear at this time why EMG is less in water, but it can be speculated that differences in muscle activity are related to reflex and/or fluid changes caused by water immersion [9]. In a study monitoring sEMG signals with isometric contractions both on land and in water, the authors summarised that the sEMG and force were not considerably influenced by the environment. The outcomes achieved in this study could be helpful to describe the functional movement of the STS task in water to aid clinical decision making in aquatic rehabilitation programs [16]. In another study looking at knee muscle isometric activity, no differences in force output were found but with reduced muscle activity via sEMG [31]. Other study describes the functional movement of the STS task in water as aquatic rehabilitation programs. It showed less muscle activity in the lower limb might allow successful completion of the STS movement for people with reduced leg strength but it should be considered higher trunk activity to control the movement in [11].

The major concern in the main methodology for measuring EMG in water is waterproofing EMG wires. The two general approaches to measure muscle activity using surface EMG during locomotion water have been the following, to create a waterproof seal located around the cables and create a waterproof system throughout the body by subjects wearing a dry suit. The overcoming of all barriers and limitations of sEMG in water worthwhile because knowledge of muscle activity is fundamental to understanding the neuromuscular responses in locomotion in water. On a review of the literature, it demonstrates that the measurement of muscle activity during locomotion in water is a surfacing area of research [9].

### Limitations

The primary limitation of this review is that all of the included studies were cross-sectional. However this review did not seek to determine the effectiveness of an intervention, for which a randomized-controlled design would be more appropriate. Also we did not search for any unpublished literature in this area and so it is possible that relevant studies may have been missed. Finally the findings of this review are based on a limited number of studies, the majority of which used a small sample size.

## Conclusion

A summary of the quantification of muscle activity during different exercises and activities in water has been discussed. In general, muscle activity tends to be lower in water-based exercises compared to land-based ones; however more research is needed to understand why.

## Competing interests

The authors declare that they have no competing interests.

## Authors’ contributions

AIC-V conceived the study, carried out the acquisition, analysis and interpretation of data. CLC-H carried out the acquisition, analysis and interpretation of data and drafted the manuscript. Both authors read and approved the final manuscript.

## Pre-publication history

The pre-publication history for this paper can be accessed here:

http://www.biomedcentral.com/2052-1847/6/15/prepub
